# Low skeletal muscle mass is associated with arterial stiffness in community-dwelling Chinese aged 45 years and older

**DOI:** 10.1186/s12889-020-8323-7

**Published:** 2020-02-13

**Authors:** Mingzhe Yang, Xuguang Zhang, Zhenkai Ding, Feijie Wang, Yufang Wang, Changya Jiao, Jie-Hua Chen

**Affiliations:** 1 Nutrition and Health Research Centre, By-Health Co. Ltd, No. 3 Kehui 3rd Street, No. 99 Kexue Avenue Central, Science City, Huangpu District, Guangzhou, 510633 China; 2 Department of Nutrition and Food Hygiene, School of Public Health, Southern Medical University, Guangzhou North Avenue 1838, Guangzhou, 510515 People’s Republic of China

**Keywords:** Skeletal muscle mass, Body composition, Pulse wave velocity, Arterial stiffness

## Abstract

**Background:**

Evidence suggests that body composition has impact on arterial stiffness. However, evidence in Chinese are limited, and results remain controversial. The aim of our study is to investigate whether skeletal muscle mass is associated with arterial stiffness in Chinese community-dwelling men and women aged 45 years and older.

**Methods:**

In this cross-sectional study, 20,477 participants (age range: 45–80 years, 68.8% women) were included in the analysis. Brachial-ankle pulse wave velocity (baPWV), an indicator of arterial stiffness was measured using a waveform device. Total muscle mass and muscle mass of arm, leg and trunk were measured by bioelectrical impedance analysis. Height and weight were measured and appendicular skeletal muscle mass index (ASMI) was calculated as appendicular skeletal muscle mass (sum of arm and leg muscle mass) divided by height square.

**Results:**

After adjustment for age, body fat percentage, systolic blood pressure and diastolic blood pressure, ASMI was negatively associated with baPWV [β (SE) for men: − 0.208 (0.016), *p* < 0.0001; for women: − 0.245 (0.012), *p* < 0.0001]. High ASMI was a protective factor for the presence of arterial stiffness (defined as baPWV) [OR (95%CI) for men: 0.730 (0.682, 0.782), *p* < 0.0001; women: 0.634 (0.593, 0.677), *p* < 0.0001]. Similar associations were found between quantity of muscle mass (total and appendicular muscle mass, muscle mass of arm, leg and trunk) and arterial stiffness in men and women after further adjustment for height (all *p* < 0.0001).

**Conclusion:**

Low skeletal muscle mass is associated with increased risk of arterial stiffness in Chinese community-dwelling adults aged 45 years and older.

## Background

Cardiovascular disease (CVD) remains to be the top cause of death in China and brings out huge medical and financial burden [1]. Arterial stiffness, a reflection of health status of both central and peripheral arteries, has strong predictive value for future cardiovascular events and all-cause mortality [2, 3]. Pulse wave velocity (PWV), a standard and noninvasive indicator applied in many epidemiological and clinical studies for assessing arterial stiffness, has been confirmed as an independent predictor of future cardiovascular events [4–8]. Results from meta-analysis showed that every 1 SD increase of the brachial-ankle pulse wave velocity (baPWV) was associated with a 1.19-fold increase in the risk of CVD [4].

Evidence suggests that body composition has impact on the prevalence and development of arterial stiffness [9–14]. More specifically, previous studies which explored the association between muscle mass and PWV showed consensus preliminarily. Significant negative associations were found between muscle mass indices and PWV in studies among apparently healthy elderly of Chinese [15], Japanese [16, 17] and American with multi-race [18]. Similar results were found among patients of type 2 diabetes [19] and peritoneal dialysis [20]. However, evidence in Chinese population are limited, and results remain controversial among different gender and for regional muscle mass (arm, leg and trunk).

Possible pathways for the association between low muscle mass and arterial stiffness might lie in insulin resistance and chronic inflammation. Loss of muscle mass was considered as a cause of insulin resistance by impairing glucose metabolism [21], and evidence showed that insulin resistance could determine the arterial elastic properties [22]. On the other hand, it had been suggested that some inflammatory cytokines were associated with an increased risk of sarcopenia [23], and some inflammatory markers had been examined as causal agents in the pathogenesis of arterial stiffness [24].

The aim of this study was to explore the associations between muscle mass indices (total and appendicular muscle mass, appendicular skeletal muscle mass index, and muscle mass of arm, leg and trunk) and arterial stiffness (defined as baPWV) in community-dwelling Chinese men and women aged 45 years and older.

## Methods

### Study design and study population

Data of the participants were from the Screening of Chronic Diseases in Chinese Adults (SCDCA) study, a cross-sectional study started in 2017, which aims to screen four chronic diseases (obesity, osteoporosis, arterial stiffness and age-related macular degeneration) and explore the influencing factors among Chinese community-dwelling adults in urban areas from 24 administrative regions. According to the population size and cooperation, we included several cities/areas in each administrative region. In each city/town, the communities for screening were enrolled using cluster random sampling. The research staff were divided into five groups, each group was equipped with mobile health check-up vehicle, a specially designed truck carried research instruments to designated sampling cities. Eligible participants in this study were men and women: 1) aged above 18 years old, 2) who have resided locally for more than 5 years, 3) who were cooperative and volunteered. The following participants were excluded: 1) pregnant and lactating women, 2) participants with major organ diseases, including heart, liver or kidney disease, 3) participants with mental diseases, 4) unable to carry out body examination due to disabilities. In the present analysis, participants from March 2017 to May 2018 were enrolled and the following were further excluded: 1) aged under 45 years, 1) participants with peripheral artery disease whose ankle-brachial index (ABI) ≤ 0.9 [25], 2) participants with incomplete information of body composition, arterial stiffness and covariates. Thus, a total of 20,477 participants were included in the present analysis. The study was approved by Ethics Committee of Chinese Clinical Trial Registry. All participants gave written informed consent.

### Anthropometric measurements

Height and weight were measured by trained medical workers according to standard procedures, with subjects wearing light indoor clothing and barefoot. Height was measured to the nearest 0.1 cm and weight was measured to the nearest 0.1 kg. BMI (body mass index) was calculated as weight (kg) divided by height squared (m^2^).

### Measurements of body composition indices

The quantity of total skeletal muscle mass (SMM), muscle mass of the left arm (LAMM), right arm (RAMM), left leg (LLMM), right leg (RLMM) and trunk (TMM) and the percentage of body fat (BFP) were measured using a dual bioelectrical impedance analyzer (IOI 353; Jawon, Korea). Bioelectrical impedance analysis (BIA) is widely used in the detection of body composition and this instrument were used in several studies [26, 27]. Appendicular skeletal muscle mass (ASM, kg) was defined as the sum of the arm and leg muscle mass on both sides. Appendicular skeletal muscle mass index (ASMI, kg/m^2^) was calculated as ASM divided by height square.

### Measurements of arterial stiffness indices and blood pressure

In this study, the baPWV, mean arterial pressure (MAP), pulse pressure (PP), upstroke time (UT) as well as the brachial and ankle blood pressures on the left and right sides of the subjects were measured with a plethysmography apparatus (BP-203RPE III; Omron, Japan) after rested in a supine position for 5 min. Evidence showed that the validity and reproducibility of this instrument is considerably high and suitable for screening vascular damages in a large population [28]. baPWV, MAP, PP and UT were calculated as the mean of the left and right baPWV, MAP and UT values, respectively. Systolic blood pressure (SBP) and diastolic blood pressure (DBP) were calculated as the mean of the left and right brachial systolic and diastolic pressure, respectively. High baPWV (arterial stiffness) was defined as baPWV ≥1800 mm/s [29].

### Data collection of other characteristics

A standard questionnaire was used to collect each participant’s age, gender, education level, medical history, and habits of smoking, drinking and exercise. Participants filled the questionnaire on a computer/smartphone under the supervision of trained research staff.

### Statistical analysis

Categorical variables were described as number and percentage. Continuous variables with normal distribution were expressed using mean with standard deviation (SD), and non-normally distributed continuous variables were presented as median with 25th and 75th percentile. The participants were stratified into three groups by tertiles of the ASMI (men: 7.71 ± 0.37 kg/m^2^, 8.53 ± 0.22 kg/m^2^, 9.78 ± 0.73 kg/m^2^; women: 6.77 ± 0.34 kg/m^2^, 7.51 ± 0.18 kg/m^2^, 8.41 ± 0.57 kg/m^2^). Differences between groups were tested using Student’s t-test or one-way ANOVA analysis for normally distributed continuous variables and Kruskal–Wallis test for continuous variables with non-normal distribution. Pearson’s correlation analysis was used to access the correlation between muscle mass indices (ASMI, ASM, SMM, LAMM, RAMM, LLMM, RLMM and TMM) and baPWV. The association of muscle mass indices with baPWV and the association of mass indices with arterial stiffness (normal / high, defined as baPWV) were carried out by multivariate linear regression analysis and multiple logistic regression analysis, respectively. The regression models testing ASMI with baPWV / arterial stiffness were performed after adjusting for age, body fat, SBP and DBP. The models testing quantity of muscle mass (SMM, ASM, LAMM, RAMM, LLMM, RLMM, TMM) with baPWV / arterial stiffness were further adjusted for body height. All analyses were conducted using SAS version 9.1 (SAS Institute, Inc., Cary, NC, USA). A *p* value < 0.05 was considered statistically significant.

## Results

The mean age of participants included in the present analysis was 61.25 ± 8.22 years (age range: 45–80 years), 68.79% were women. Compared with women, men were older and had higher BMI, SMM, AMM, LAMM, RAMM, LLMM, RLMM, TMM, baPWV, SBP, DBP and ABI, however, their BFP, PP, MAP and UT were lower than that of women. Compared with women, the prevalence of diabetes and hypertension, the percentage of high education level, current smoker, current alcohol user and regular exercise were higher in men (all *p* < 0.05) (Table 1).

**Table 1:** Characteristics of the study population by gender

	Men	Women	*p*^a^
N	6390	14,087	
Age (years)	62.87 ± 8.07	60.30 ± 8.00	< 0.0001
High education level (%)	14.76	11.05	< 0.0001
Current smokers (%)	31.84	2.27	< 0.0001
Current alcohol user (%)	40.30	8.03	< 0.0001
Regular exercise (%)	20.09	18.41	< 0.0001
Type 2 diabetes (%)	11.07	9.86	< 0.0001
Hypertension (%)	30.76	26.65	< 0.0001
Height (cm)	168.6 ± 5.88	158.24 ± 5.35	< 0.0001
Weight (kg)	69.78 ± 10.52	61.13 ± 9.35	< 0.0001
BMI (kg/m^2^)	24.51 ± 3.23	24.39 ± 3.40	0.014
SMM (kg)	49.36 ± 6.18	38.13 ± 4.32	< 0.0001
LAMM (kg)	3.30 ± 0.48	2.50 ± 0.33	< 0.0001
RAMM (kg)	3.31 ± 0.49	2.49 ± 0.35	< 0.0001
LLMM (kg)	9.05 ± 1.28	7.00 ± 0.93	< 0.0001
RLMM (kg)	9.06 ± 1.27	6.99 ± 0.90	< 0.0001
TMM (kg)	24.65 ± 2.78	19.16 ± 1.96	< 0.0001
ASM (kg)	24.72 ± 3.47	18.98 ± 2.42	< 0.0001
ASMI (kg/m^2^)	8.68 ± 0.98	7.57 ± 0.78	< 0.0001
BFP (%)	23.50 (19.70, 26.90)	31.80 (28.50, 34.70)	< 0.0001
baPWV (cm/s)	1650.00 (1449.00, 1894.00)	1567.00 (1372.00, 1822.00)	< 0.0001
SBP (mmHg)	137.41 ± 19.00	135.87 ± 20.63	< 0.0001
DBP (mmHg)	81.32 ± 10.95	78.34 ± 11.14	< 0.0001
PP (mmHg)	58.00 ± 12.19	59.58 ± 13.13	< 0.0001
ABI	1.21 ± 0.012	1.18 ± 0.10	< 0.0001
MAP (%)	39.85 ± 3.93	42.50 ± 3.46	< 0.0001
UT (s)	144.00 (133.00, 160.00)	156.00 (142.00, 174.00)	< 0.0001

Values are percentage, mean ± SD or medians (25th and 75th percentile)

*Abbreviations: BMI* Body mass index, *SMM* Total muscle mass, *LAMM* Left arm muscle mass, *RAMM* Right arm muscle mass, *LLMM* Left arm muscle mass, *RLMM* Right leg muscle mass, *TMM* Trunk muscle mass, *BFP* total body fat percentage, *ASM* Appendicular skeletal muscle, *ASMI* Appendicular skeletal muscle index, *baPWV* Brachial-ankle pulse wave velocity, *SBP* Systolic blood pressure, *DBP* Diastolic blood pressure, *PP* Pulse pressure, *ABI* Ankle brachial index, *MAP* Mean artery pressure, *UT* Upstroke time

^a^Test for difference between gender groups was performed using the Chi-square test for categorical variables, Kruskal–Wallis test for non-normally distributed continuous variables and T test for normally distributed continuous variables

Characteristics of body composition and artery stiffness in men and women are presented in Table 2. The participants were divided into three groups according to ASMI tertiles (men: 7.71 ± 0.37 kg/m^2^, 8.53 ± 0.22 kg/m^2^, 9.78 ± 0.73 kg/m^2^; women: 6.77 ± 0.34 kg/m^2^, 7.51 ± 0.18 kg/m^2^, 8.41 ± 0.57 kg/m^2^). Both among men and women, higher ASMI was observed in participants who were younger and those with lower baPWV and MAP (all *p* < 0.0001). In contrast, participants with higher ASMI had higher BMI, ASM, SMM, LAMM, RAMM, LLMM, RLMM, TMM, SBP, DBP, PP, ABI and UT (all *p* < 0.05). The highest BFP was found in median ASMI group (T2) for men and in high ASMI group (T3) for women.

**Table 2:** Characteristics of the study population according to ASMI tertiles

	ASMI			*p*^a^
	T1	T2	T3	
Men (*n* = 6390)				
Age (years)	64.62 ± 8.28	62.73 ± 7.94	61.86 ± 8.17	< 0.0001
Height (cm)	167.71 ± 5.78	168.68 ± 5.91	169.42 ± 5.81	< 0.0001
Weight (kg)	60.67 ± 6.86	70.33 ± 6.92	78.34 ± 8.97	< 0.0001
BMI (kg/m^2^)	21.55 ± 2.05	24.71 ± 1.97	27.27 ± 2.60	< 0.0001
ASMI (kg/m^2^)	7.71 ± 0.37	8.53 ± 0.22	9.78 ± 0.73	< 0.0001
ASM (kg)	21.71 ± 1.87	24.31 ± 1.83	28.12 ± 2.90	< 0.0001
SMM (kg)	44.30 ± 3.66	48.70 ± 3.71	55.09 ± 5.34	< 0.0001
LAMM (kg)	2.89 ± 0.26	3.25 ± 0.26	3.77 ± 0.41	< 0.0001
RAMM (kg)	2.89 ± 0.27	3.25 ± 0.27	3.77 ± 0.44	< 0.0001
LLMM (kg)	7.96 ± 0.70	8.90 ± 0.69	10.29 ± 1.11	< 0.0001
RLMM (kg)	7.97 ± 0.69	8.91 ± 0.68	10.29 ± 1.08	< 0.0001
TMM (kg)	22.59 ± 1.88	24.39 ± 1.94	26.96 ± 2.51	< 0.0001
BFP (%)	21.10 (17.40, 24.40)	25.10 (22.00, 28.00)	23.90 (20.10, 27.80)	< 0.0001
baPWV (cm/s)	1677.00 (1459.00, 1945.00)	1647.00 (1451.00, 1876.00)	1629.00 (1434.00, 1854.00)	< 0.0001
SBP (mmHg)	134.35 ± 18.81	137.39 ± 18.14	140.50 ± 19.52	< 0.0001
DBP (mmHg)	79.16 ± 10.44	81.48 ± 10.70	83.31 ± 11.30	< 0.0001
PP (mmHg)	57.18 ± 12.40	57.79 ± 11.92	59.03 ± 12.18	< 0.0001
ABI	1.19 ± 0.11	1.21 ± 0.12	1.23 ± 0.12	< 0.0001
MAP (%)	40.14 ± 3.80	39.74 ± 3.94	39.66 ± 4.02	< 0.0001
UT (s)	143.50 (132.00, 160.00)	144.00 (132.00, 159.00)	145.00 (134.00, 161.00)	0.0238
Women (*n* = 14,087)				
Age (years)	61.53 ± 8.26	60.16 ± 8.06	59.60 ± 7.84	< 0.0001
Height (cm)	157.73 ± 5.51	158.38 ± 5.24	158.63 ± 5.26	< 0.0001
Weight (kg)	53.07 ± 5.93	60.74 ± 5.52	69.57 ± 7.74	< 0.0001
BMI (kg/m^2^)	21.32 ± 2.05	24.21 ± 1.83	27.64 ± 2.66	< 0.0001
ASMI (kg/m^2^)	6.77 ± 0.34	7.51 ± 0.18	8.41 ± 0.57	< 0.0001
ASM (kg)	16.87 ± 1.51	18.87 ± 1.33	21.19 ± 2.05	< 0.0001
SMM (kg)	34.63 ± 2.96	37.96 ± 2.68	41.81 ± 3.78	< 0.0001
LAMM (kg)	2.21 ± 0.21	2.48 ± 0.19	2.79 ± 0.29	< 0.0001
RAMM (kg)	2.21 ± 0.22	2.48 ± 0.20	2.79 ± 0.32	< 0.0001
LLMM (kg)	6.23 ± 0.58	6.96 ± 0.53	7.82 ± 0.84	< 0.0001
RLMM (kg)	6.22 ± 0.56	6.95 ± 0.51	7.79 ± 0.78	< 0.0001
TMM (kg)	17.75 ± 1.51	19.10 ± 1.40	20.62 ± 1.79	< 0.0001
BFP (%)	29.10 (26.00, 31.90)	31.90 (29.10, 34.30)	34.30 (31.50, 36.80)	< 0.0001
baPWV (cm/s)	1587.00 (1375.50, 1867.00)	1558.00 (1362.00, 1812.00)	1557.00 (1378.00, 1789.00)	< 0.0001
SBP (mmHg)	132.40 ± 20.17	135.19 ± 20.55	140.02 ± 20.44	< 0.0001
DBP (mmHg)	76.45 ± 11.01	78.01 ± 11.03	80.57 ± 11.00	< 0.0001
PP (mmHg)	57.99 ± 12.75	59.16 ± 13.06	61.58 ± 13.31	< 0.0001
ABI	1.16 ± 0.10	1.18 ± 0.10	1.19 ± 0.11	< 0.0001
MAP (%)	42.70 ± 3.43	42.53 ± 3.40	42.25 ± 3.55	< 0.0001
UT (s)	155.00 (140.00, 173.00)	156.00 (142.00, 174.00)	158.00 (143.00, 176.00)	< 0.0001

Values are mean ± SD or medians (25th and 75th percentile)

*Abbreviations*: *BMI* Body mass index, *SMM* Total muscle mass, *LAMM* Left arm muscle mass, *RAMM* Right arm muscle mass, *LLMM* Left arm muscle mass, *RLMM* Right leg muscle mass, *TMM* Trunk muscle mass, *BFP* total body fat percentage, *ASM* Appendicular skeletal muscle, *ASMI* Appendicular skeletal muscle index, *baPWV* Brachial-ankle pulse wave velocity, *SBP* systolic blood pressure, *DBP* diastolic blood pressure, *PP* Pulse pressure, *ABI* Ankle brachial index, *MAP* Mean artery pressure, *UT* Upstroke time

^a^Test for difference between tertiles was performed using the Kruskal–Wallis test for non-normally distributed continuous variables and ANOVA analysis for normally distributed continuous variables

Pearson’s correlations between muscle mass indices and baPWV are shown in Table 3. Significant negative correlations were observed between muscle mass indices (ASMI, ASM, SMM, LAMM, RAMM, LLMM, RLMM, and TMM) and baPWV in men and women (all *p* < 0.0001).

**Table 3:** Correlation between muscle mass indices and baPWV

	Men (*n* = 6390)		Women (*n* = 14,087)	
	r	*p*	r	*p*
ASMI (kg/m^2^)	− 0.080	< 0.0001	− 0.054	< 0.0001
ASM (kg)	− 0.115	< 0.0001	− 0.112	< 0.0001
SMM (kg)	− 0.120	< 0.0001	− 0.114	< 0.0001
LAMM (kg)	− 0.106	< 0.0001	− 0.116	< 0.0001
RAMM (kg)	− 0.107	< 0.0001	− 0.115	< 0.0001
LLMM (kg)	−0.114	< 0.0001	− 0.100	< 0.0001
RLMM (kg)	−0.116	< 0.0001	− 0.111	< 0.0001
TMM (kg)	−0.123	< 0.0001	− 0.114	< 0.0001

Pearson’s correlation coefficient was calculated using bivariate correlation analysis

The natural logarithm was applied for baPWV

*Abbreviations*: *baPWV* Brachial-ankle pulse wave velocity, *ASMI* appendicular skeletal muscle index, *ASM* appendicular skeletal muscle, *SMM* Total muscle mass, *LAMM* Left arm muscle mass, *RAMM* Right arm muscle mass, *LLMM* Left arm muscle mass, *RLMM* Right leg muscle mass, *TMM* Trunk muscle mass

The results of the multiple linear regression showed that ASMI was negatively associated with baPWV [β (SE) for men: − 0.208 (0.016), *p* < 0.0001; women: − 0.245 (0.012), *p* < 0.0001] adjusted for age, body fat percentage, systolic blood pressure and diastolic blood pressure. Similar associations were found between quantity of muscle mass (ASM, SMM, LAMM, RAMM, LLMM, RLMM, and TMM) and baPWV in men and women after further adjustment for height (all *p* < 0.0001) (Table 4).

**Table 4:** Multiple linear regression for association between muscle mass indices and baPWV adjusted for potential confounders

	Model 1			Model 2		
	R^2^	β (SE)	*p*	R^2^	β (SE)	*p*
Men						
ASMI (kg/m^2^)	0.172	−0.040 (0.020)	0.0431	0.471	−0.208 (0.016)	< 0.0001
ASM (kg)	0.173	−0.061 (0.016)	0.0002	0.473	−0.200 (0.016)	< 0.0001
SMM (kg)	0.172	−0.053 (0.018)	0.0033	0.472	−0.232 (0.020)	< 0.0001
LAMM (kg)	0.173	−0.062 (0.015)	< 0.0001	0.471	−0.176 (0.015)	< 0.0001
RAMM (kg)	0.174	−0.062 (0.015)	< 0.0001	0.471	−0.164 (0.015)	< 0.0001
LLMM (kg)	0.173	−0.057 (0.016)	0.0003	0.473	−0.198 (0.016)	< 0.0001
RLMM (kg)	0.173	−0.058 (0.016)	0.0003	0.472	−0.198 (0.017)	< 0.0001
TMM (kg)	0.172	−0.037 (0.020)	0.0676	0.469	−0.255 (0.025)	< 0.0001
Women						
ASMI (kg/m^2^)	0.288	0.001 (0.014)	0.9294	0.579	−0.245 (0.012)	< 0.0001
ASM (kg)	0.288	−0.026 (0.012)	0.0266	0.579	−0.206 (0.013)	< 0.0001
SMM (kg)	0.288	−0.006 (0.013)	0.6521	0.578	−0.244 (0.016)	< 0.0001
LAMM (kg)	0.288	−0.041 (0.011)	0.0002	0.579	−0.178 (0.011)	< 0.0001
RAMM (kg)	0.288	−0.040 (0.011)	0.0002	0.577	−0.155 (0.011)	< 0.0001
LLMM (kg)	0.288	−0.017 (0.011)	0.1317	0.578	−0.187 (0.012)	< 0.0001
RLMM (kg)	0.288	−0.022 (0.012)	0.0594	0.579	−0.203 (0.013)	< 0.0001
TMM (kg)	0.288	0.025 (0.015)	0.0858	0.576	−0.259 (0.020)	< 0.0001

Multiple linear regression. Model 1: adjusted for age. Model 2: further adjusted for BFP, SBP and DBP for the association between ASMI and baPWV, further adjusted for height, BFP, SBP and DBP for the association between ASM, SMM, LAMM, RAMM, LLMM, RLMM, TMM and baPWV

*Abbreviations*: *baPWV* Brachial-ankle pulse wave velocity, *SMM* Total muscle mass, *ASMI* Appendicular skeletal muscle index, *ASM* Appendicular skeletal muscle, *LAMM* Left arm muscle mass, *RAMM* Right arm muscle mass, *LLMM* Left arm muscle mass, *RLMM* Right leg muscle mass, *TMM* Trunk muscle mass

Multiple logistic regression analysis showed that higher ASMI was a protective factor for the presence of arterial stiffness adjusted for age, body fat percentage, systolic blood pressure and diastolic blood pressure [OR (95%CI) for men: 0.730 (0.682, 0.782), *p* < 0.0001; women: 0.634 (0.593, 0.677), *p* < 0.0001]. Similar associations were found between quantity of muscle mass (ASM, SMM, LAMM, RAMM, LLMM, RLMM, and TMM) and arterial stiffness in men and women after further adjustment for height (all *p* < 0.0001) (Table 5).

**Table 5:** Multiple logistic regression for the association between muscle mass indices and arterial stiffness (0: baPWV≤1800, 1: baPWV> 1800) adjusted for potential confounders

	Model 1		Model 2	
	OR (95% CI)	*p*	OR (95% CI)	*p*
Men				
ASMI (kg/m^2^)	0.942 (0.889, 0.997)	0.0391	0.731 (0.683, 0.783)	< 0.0001
ASM (kg)	0.976 (0.960, 0.992)	0.0033	0.899 (0.878, 0.921)	< 0.0001
SMM (kg)	0.989 (0.980, 0.998)	0.0220	0.941 (0.927, 0.955)	< 0.0001
LAMM (kg)	0.818 (0.727, 0.919)	0.0007	0.480 (0.406, 0.568)	< 0.0001
RAMM (kg)	0.819 (0.731, 0.919)	0.0006	0.510 (0.433, 0.599)	< 0.0001
LLMM (kg)	0.941 (0.900, 0.983)	0.0067	0.755 (0.709, 0.805)	< 0.0001
RLMM (kg)	0.941 (0.900, 0.984)	0.0078	0.757 (0.709, 0.808)	< 0.0001
TMM (kg)	0.985 (0.965, 1.006)	0.1593	0.876 (0.844, 0.908)	< 0.0001
Women				
ASMI (kg/m^2^)	0.949 (0.899, 1.001)	0.0551	0.634 (0.594, 0.677)	< 0.0001
ASM (kg)	0.974 (0.957, 0.992)	0.0037	0.844 (0.820, 0.869)	< 0.0001
SMM (kg)	0.992 (0.982, 1.002)	0.1065	0.900 (0.886, 0.914)	< 0.0001
LAMM (kg)	0.794 (0.699, 0.902)	0.0004	0.348 (0.286, 0.425)	< 0.0001
RAMM (kg)	0.807 (0.714, 0.914)	0.0007	0.397 (0.329, 0.480)	< 0.0001
LLMM (kg)	0.946 (0.903, 0.990)	0.0158	0.668 (0.621, 0.719)	< 0.0001
RLMM (kg)	0.937 (0.893, 0.982)	0.0068	0.616 (0.573, 0.661)	< 0.0001
TMM (kg)	1.001 (0.979, 1.023)	0.9587	0.795 (0.766, 0.826)	< 0.0001

Multiple logistic regression. Model 1: adjusted for age. Model 2: further adjusted for BFP, SBP and DBP for the association between ASMI and baPWV, further adjusted for height, BFP, SBP and DBP for the association between ASM, SMM, LAMM, RAMM, LLMM, RLMM, TMM and baPWV

*Abbreviations*: *baPWV* Brachial-ankle pulse wave velocity, *SMM* Total muscle mass, *ASMI* Appendicular skeletal muscle index, *ASM* Appendicular skeletal muscle, *LAMM* Left arm muscle mass, *RAMM* Right arm muscle mass, *LLMM* Left arm muscle mass, *RLMM* Right leg muscle mass, *TMM* Trunk muscle mass

## Discussion

The present study found that ASMI and quantity of muscle mass (total and appendicular muscle mass, muscle mass of arm, leg and trunk) were negatively associated with baPWV. High levels of these muscle mass indices were protective factors for the presence of arterial stiffness in Chinese community-dwelling men and women aged 45 years and older.

Recently, epidemiological data showed that the middle-aged population in North China had a high prevalence of arterial stiffness (high baPWV) [30]. Compared with this study, the prevalence of arterial stiffness in the present analysis was higher in men and approximate in women (men: 33.43%, women: 26.63%, data not shown). Meanwhile, the quantity of total skeletal muscle mass of Chinese adults was at a low level, especially for the elderly [31], which was comparable with our results.

The negative associations between SMM / ASM / ASMI and baPWV were found in previous studies [15, 17, 20]. Zhang et al. [15] found that ASMI was associated with baPWV in 1002 Chinese community dwelling participants aged 65 years and above, and this relation remains significant only in men in analysis of gender subgroup. Similar association was observed between total skeletal muscle mass and baPWV among Chinese peritoneal dialysis patients [20], which was mainly contributed by female patients. Moreover, studies among Japanese women suggested that baPWV was a risk factor for the low level of ASMI [17]. In accordance with these studies, our results suggested that Chinese adults with low levels of SMM / ASM / ASMI had higher baPWV. Consistent results were observed both in men and women. Our findings from a large sample of Chinese community-dwelling population confirmed the hypothesis that low level of muscle mass is a potential risk factor of arterial stiffness. To our knowledge, this relation was proved in both genders for the first time. Possible reasons for the conflicting results between men and women included that: 1) the level of baPWV, muscle mass or other covariates were significantly different between gender groups, 2) the sample size in the subgroup was limited for examining the potential associations, 3) some androgens [32, 33] and the different distribution of body composition in men and women [34, 35] might have potential effect on this relation.

Several studies further explored the impact of regional muscle mass quantity on pulse wave velocity which showed controversial results. A study among multi-race American elderly reported that high cfPWV (carotid-femoral pulse wave velocity) was associated with lower leg and arm mass in all men and lower leg mass in white women [18]. However, among Asian Indians with diabetes, negative correlation was observed between cfPWV and fat free mass in left leg in all, right arm, left arm, truncal fat free mass in men and right leg fat free mass in women [19]. In addition, mid-thigh muscle cross-sectional area was found to be an independent determinant of baPWV in middle-aged to elderly Japanese men [16]. Our study firstly found that arm and leg muscle mass on both sides and trunk muscle mass were both negatively associated with baPWV in Chinese community-dwelling men and women aged 45 years and older. Reasons for the controversial results may lie in: 1) the different indicators used for assessing arterial stiffness: cfPWV reflects central arterial stiffness and baPWV reflects both central and peripheral arterial stiffness [36], 2) age-related muscle mass decline (onset of sarcopenia), which is mainly characterized by the loss of appendicular muscle mass [37]. More studies are needed to confirm the associations between muscle mass quantities of different regions and arterial stiffness.

Although previous studies reported liner association between muscle mass indices and baPWV, to our knowledge, this was the first study performed multiple logistic regression analysis to examine the potential effect of muscle mass indices on arterial stiffness (baPWV as the dependent variable). Since arterial stiffness is a predictor for future cardiovascular disease [3], this analysis showed much clinical value. Our study found that the ASMI increased by 0.98 and 0.78 kg/m^2^, the risks of developing arterial stiffness decreased by 27.0 and 36.6% in men and women, respectively. The indices of muscle mass quantity increased by 1 SD, the risks of developing arterial stiffness decreased from 5.9% (SMM) to 52.0% (LAMM) in men and from 10.0% (SMM) to 65.2% (LAMM) in women. The effect of arm muscle mass quantity on risk of arterial stiffness was more pronounced.

It is well known that CVD brings out huge medical and financial burden because of its high mortality [1]. Arterial stiffness, a predictor of future CVD events [3], shows a high prevalence in middle-aged Chinese [30]. Meanwhile, the quantity of total skeletal muscle mass of Chinese adults was at a low level [31]. The results above suggested that participants with higher muscle mass quantity have low risk for developing arterial stiffness, which indicated that taking actions to preserve muscle mass might have potential benefits for prevention of cardiovascular disease. More specific, the findings indicated that: 1) not only men but also women should take actions to preserve muscle mass; 2) muscle loss should be monitored at an earlier age of 45 rather than the elderly; 3) medical staff in community should pay more attention on arm muscle mass in solutions for maintaining muscle quantity such as resistance training. The possible influences of our findings on programmatic interventions of public health are as follows: Among Chinese community residents aged 45 years and older, 1) quantity of muscle mass should be regularly screened, since loss of muscle mass is difficult to be perceived without obvious decline of muscle function, 2) more clinical or community programs for improving level of muscle mass should be implemented such as some courses of nutrition and physical training.

Several mechanisms may involve in the association between muscle mass and arterial stiffness. Firstly, loss of muscle mass was considered as a cause of insulin resistance by impairing glucose metabolism [21]. Evidence suggested that insulin resistance could determine the arterial elastic properties and coronary flow reserve [22] and played an important role in the development of atherosclerotic cardiovascular disease [38]. Secondly, chronic inflammation was widely studied as a pathway both to muscle loss and arterial stiffness. Results from a cohort study in Chinese sarcopenia patients showed that high levels of the inflammatory cytokines TWEAK and TNF-α were associated with an increased risk of sarcopenia, while the insulin growth factor 1, insulin, and adiponectin were associated with a decreased risk of sarcopenia [23]. Meanwhile, these mediators of inflammation had been examined as biomarkers and causal agents in the pathogenesis of arterial stiffness [24] and atherosclerosis, and they had been considered as a therapeutic target for cardiovascular disease [39]. These studies supported that loss of muscle mass might be a risk factor of arterial stiffness and further affected the development of CVD. However, experimental and clinical studies are warranted.

There are some limitations in the present study. First, due to the cross-sectional design of our study, we cannot make inferences about causality which warrants further prospective and interventional researches. Secondly, bioelectrical impedance analysis (BIA) but not Dual energy X-ray absorptiometry (DEXA, considered as gold standard) was used to measure body composition. However, evidence have shown that body composition measured by BIA were strongly correlated with that measured by DEXA [40] and have been widely used in epidemiological and clinical studies [31, 41]. In addition, compared with DEXA, BIA can avoid the risk of exposure to ionized radiation and be more practical and economical for studies with a large sample. Finally, the impact of blood biomarkers were not evaluated which evidences have shown to be associated with arterial stiffness [42–44].

## Conclusion

In conclusion, our study indicated that low muscle mass was associated with increased risk of arterial stiffness in Chinese community-dwelling adults aged 45 years and older. Further longitudinal researches are needed to confirm the potential effects of muscle mass on arterial stiffness observed in this study for the prevention of cardiovascular disease.

## Data Availability

The datasets generated and/or analyzed during the current study are not publicly available due the government policy for original Chinese biodata of Chinese population but are available from the corresponding author on reasonable request.

## Abbreviations

ABI: Ankle brachial index
ASM: Appendicular skeletal muscle mass
ASMI: Appendicular skeletal muscle mass index
baPWV: Brachial-ankle pulse wave velocity
BFP: Percentage of body fat
BIA: Bioelectrical impedance analysis
BMI: Body mass index
CfPWV: Carotid-femoral pulse wave velocity
CVD: Cardiovascular disease
DBP: Diastolic blood pressure
DEXA: Dual energy X-ray absorptiometry
LAMM: Muscle mass of the left arm
LLMM: Muscle mass of left leg
MAP: Mean artery pressure
PP: Pulse pressure
PWV: Pulse wave velocity
RAMM: Muscle mass of right arm
RLMM: Muscle mass of right leg
SBP: Systolic blood pressure
SD: Standard deviation
SMM: Total skeletal muscle mass
TMM: Muscle mass of trunk
UT: Upstroke time
